# Scientific Items

**Published:** 1880-08-20

**Authors:** 


### SCIENTIFIC ITEMS.

**Explosive Combinations in Pharmacy.**—We select the following items from an interesting article on dispensing in the London Chemist's and Druggist's Almanac for 1880 :

Chloride or iodide of nitrogen is formed by the addition of chlorine or a chloride, or iodine or an iodide, to ammonia; and this compound is liable to violent explosion on coming in contact with phosphorus, iodine, arsenic, olive or cod-liver oil, turpentine, etc.

Tincture of iodine and ammonia are often prescribed together, and iodide of nitrogen is necessarily produced. The rarity of accidents is due to the fact that the iodide is not free from water.

Mr. Rice, in New Remedies, mentions an explosion resulting from the preparation of the following prescription, iodide of nitrogen being evidently the cause :

℞ Iodini .................................................... gr. xv.
Lin. campho. co............................................ gr. lx.
Lin. saponis co............................................ gr. lx.

A concentrated solution of iodine and iodide of potassium was filtered through paper. The next day the filter was touched to be removed, when the paper and funnel broke into atoms with a loud explosion.

Concentrated solutions of permanganate of potash in alcohol are liable to explosion, and bichromate of potash in alcohol may ignite the latter. Aqua regia will also often cause an explosion with alcoholates or essences.

Chlorate of potash mixed dry with tannin is dangerous, and an explosion has resulted from its mixture with muriate of morphia.

The following prescription was presented at a pharmacy in New York ; it cannot be prepared without an explosion :

℞ Lactis sulphuris.......................................... gr. iij.
Antimon. sulph. aurant.................................... gr. iij.
Zinci valerian............................................ gr. j.
Potass. chlorat........................................... gr. ij.

The addition of nitrate of silver to essenee of bitter almonds to remove the hydrocyanic acid has been followed by ignition.

The following compounds have at different times caused more or less serious accidents :

℞ Calcis hypophosphitis.................................... gr. viij.
Potassæ chloratis......................................... gr. xij.
Ferri lactatis............................................ gr. v.

The trituration of hypophosphite of lime alone has sometimes resulted in an explosion. A man was killed at Erfurt while drying one kilogramme of the salt in a sand bath. It is said to be most dangerous if quite pure.

℞ Glycerini................................................ f℥ ij.
Acidi chromici............................................ ℥j.

This mixture can be made by adding the acid to the glycerin by very slow degrees.

A mixture containing chlorate of potash, tincture of perchloride of iron, and glycerine once burst in the pocket of a patient.

Pills containing oxide of silver are liable to inflame if they become warm. They have taken fire in the pocket of a customer, causing severe burns.

Other compounds liable to inflame during or after preparation are permanganate of potash and extract of milfoil, permanganate of potash and reduced iron in pills, golden sulphuret of antimony and chlorate of soda in pills.

It is always dangerous to associate glycerine or, in general, any de-oxidizer with easily-reducible compounds, such as permanganates, chromic acid, the chlorates, and some organic acid.—*Boston Journal of Chemistry.*

**Auditory Nerve.**—M. Mathias Duval, in a recent communication to the Societe de Biologie, Paris, reported the results of examinations made by him on the origin of the auditory nerve.

Several years since Prof. Cyon, of St. Petersburgh, announced that this nerve served two functions, or rather consisted of two functionally distinct nerves, one supplying the sense of hearing, and the other connected with the semi-circular canals which have long been known to have to do somewhat with the maintenance of equilibrium, supplying a new sense—the sense of space. It is also well known that the cerebellum appears to have some function connected with the equilibration of the body, vertigo being the constant symptom of its lesions, as it also is of certain disorders of the auditory apparatus, such as Meniere's disease or aural vertigo. Now, according to Mr. Duval's recent researches, the auditory nerve, the portio mollis of the seventh pair, arises from two distinct roots, one of these, the posterior, originating in the nucleus described by the majority of authors, as that for the nerve of hearing; and the other, the anterior, arising from a motor nucleus, some of the fibers, however, being traced back to the cerebellum. Putting all these facts together, M. Duval considers that the anterior root serves the sense of space, of which the semicircular canals are the peripheral organs.—*Chicago Gazette.*

**The Polyscope.**—By its use the whole mouth can readily be illuminated by the electric light without the slightest inconvenience or discomfort to the patient, and a most perfect cautery can be obtained when required for the destruction of sensitive dentine, nerves, etc.

The perfection to which this instrument has been brought was due to M. E. Brasseur and M. Troune, of Paris.—*Med. Press and Circular.*

**Bones and Sugar.**—According to the *Chemiker Zeitung*, the battle-fields of the year 1812 in Russia are still carefully sought over for bones, which are converted into bone-black. It may thus happen that a man of the present day may consume sugar which has been decolorized and purified by means of the bones of his forefathers.—*Boston Journal of Chemistry.*

